# *Plasmodium vivax* Prevalence in Semiarid Region of Northern Kenya, 2019

**DOI:** 10.3201/eid2911.230299

**Published:** 2023-11

**Authors:** Wendy Prudhomme O’Meara, Linda Maraga, Hannah Meredith, Daniel Esimit, Gilchrist Lokoel, Tabitha Chepkwony, Joseph Kipkoech, George Ambani, Diana Menya, Elizabeth Freedman, Steve Taylor, Andrew Obala

**Affiliations:** Duke University, Durham, North Carolina, USA (W. Prudhomme O’Meara, H. Meredith, E. Freedman, S. Taylor);; Moi University, Eldoret, Kenya (W. Prudhomme O’Meara, D. Menya, A. Obala);; Academic Model Providing Access to Healthcare, Eldoret (L. Maraga, T. Chepkwony, J. Kipkoech, G. Ambani);; Turkana County Health Management, Lodwar, Kenya (D. Esimit, G. Lokoel)

**Keywords:** *Plasmodium vivax*, sub-Saharan Africa, epidemiology, malaria, parasites, vector-borne infections, zoonoses, Turkana, Kenya

## Abstract

In urban and rural areas of Turkana County, Kenya, we found that 2% of household members of patients with *Plasmodium* *falciparum* infections were infected with *P*. *vivax*. Enhanced surveillance of *P*. *vivax* and increased clinical resources are needed to inform control measures and identify and manage *P*. *vivax* infections.

Until recently, little or no endemic transmission of *Plasmodium vivax* has been reported in sub-Saharan Africa outside of the Horn of Africa (*1*). *P. vivax* was presumed to be largely absent because the Duffy blood group antigen was rare in persons living in the region. However, accumulating evidence of endemic *P. vivax* has indicated that this parasite might be present in many areas of sub-Saharan Africa, albeit at low levels, and Duffy antigen–negative persons can be infected and contribute to transmission (*2*).

Turkana County is in northwestern Kenya and shares a border with Uganda, South Sudan, and Ethiopia. Turkana county’s harsh climate is characterized by an average rainfall of <215 mm/year and daytime temperatures of 40°C. Malaria transmission in this region was predicted to occur in isolated pockets with epidemic potential only after unusual rainfall. However, reactive case detection conducted across central Turkana County documented year-round symptomatic and asymptomatic *P. falciparum* infections and confirmed perennial endemic transmission of malaria (*3*). 

We hypothesized that *P. vivax* might also be circulating in Turkana County because of stable malaria transmission and proximity to Ethiopia, where *P. vivax* infections are endemic. To test this hypothesis, we extracted genomic DNA from 3,305 dried blood spots collected from household members of patients with *P. falciparum* infections; household members were enrolled in the study at their homes in catchment areas surrounding 3 rural and 3 urban health facilities in central Turkana County (*3*). The study was approved by the Moi University Institutional Research and Ethics Committee and Duke University Institutional Review Board.

We tested each DNA sample for *P. vivax* by using an established nested qualitative PCR protocol (*4*). Gel electrophoresis bands were identified independently by 2 observers. We randomly selected 15 extracts for retesting by probe-based real-time PCR with the same primer sequences to detect the same target; all PCR products were confirmed. For our analysis, we used nested qualitative PCR results. 

The percentage of household members infected with *P. vivax* was 2.1% (69/3,305); of those, 45% (31/69) were co-infected with *P. falciparum* (Table). We detected *P. vivax* infections across our study transect throughout most of the year (Figure; Appendix Figures 1, 2); the highest (5.8%, 28/485) prevalence was recorded near an urban facility in the town of Lodwar. Infections were present across all age groups, but we observed a slightly higher (1.6%, 8/490) percentage of *P*. *vivax* monoinfections in children <5 years of age (Table). Ten *P*. *vivax*–infected participants reported malaria-like symptoms when they were screened; 7 of those were co-infected with *P*. *falciparum*. Only 3 *P*. *vivax*–infected participants had a malaria-like illness within 1 month before enrollment; none reported taking antimalarial drugs. None of the *P*. *vivax*–infected participants reported traveling outside of their subcounty within 2 months before enrollment; 16% (11/69) reported having a net for their sleeping space, which was slightly less than uninfected participants (19.7%, 468/2,376) who had a net.

**Table:** *Plasmodium* *falciparum* and *P*. *vivax* infections according to age groups of household members in study of *P*. *vivax* prevalence in semiarid region of northern Kenya, 2019*

Infection type	Age range, y			
	<5, n = 490	6–15, n = 1,069	16–40, n = 1,324	>40, n = 318
Any *Plasmodium* sp.	166 (33.9)	368 (34.4)	397 (30.0)	98 (30.8)
*P*. *falciparum* only	151 (30.8)	344 (32.2)	373 (28.2)	92 (28.9)
*P*. *vivax* only	8 (1.6)	13 (1.2)	13 (0.98)	4 (1.3)
Mixed	7 (1.4)	11 (1.0)	11 (0.83)	2 (0.63)

*Values are no. (%) positive samples for each age group. Total number of samples tested was 3,305. However, 47 tested samples had missing age information, of which 20 were infected with *P*. *falciparum* and none with *P*. *vivax*; 57 samples were tested for *P*. *vivax* but not *P*. *falciparum*, of which none were positive for *P*. *vivax.* Therefore, the total number of samples in this table is 3,201. *P*. *falciparum* PCR methods and results are reported in (*3*).

**Figure:**
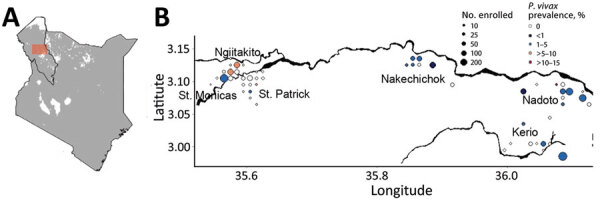
Prevalence of *Plasmodium* *vivax* infection in communities along the Turkwel River in study of *P*. *vivax* prevalence in semiarid region of northern Kenya, 2019. Household members of patients with *P*. *falciparum* infections were tested for *P*. *vivax* infection. A) Study area (red box) in Turkana County, northwestern Kenya. Gray shading indicates <0.01% prevalence of *P*. *vivax* infections; white shading indicates no detected infections. Data from the Malaria Atlas Project. B) Coordinates of different study enrollment sites. Main black line across the graph indicates the Turkwel River in Turkana County. Sizes of dots indicate number of household members enrolled; colors indicate percentages of household members who were positive for *P*. *vivax* by qualitative PCR.

The burden of *P*. *vivax* infections in sub-Saharan Africa remains unclear; infections are rarely diagnosed in a clinical setting and might often be asymptomatic. The recommended rapid diagnostic test in most countries of sub-Saharan Africa is *P*. *falciparum*–specific. Consequently, *P*. *vivax* infections might be underestimated or undocumented.

Strategies designed to eliminate *P*. *falciparum* are undermined by *P*. *vivax* because dormant *P*. *vivax* hypnozoites that can cause relapse and sustain transmission are difficult to detect and treat (*5*). Furthermore, *P*. *vivax* infections generate gametocytes before symptom onset, making detection and treatment challenging before onward transmission occurs. *P*. *vivax* infections could present a growing challenge in Kenya, even as *P*. *falciparum* is brought under control, a process that has been observed in co-endemic malaria settings in Southeast Asia (*6*).

We did not test participants for Duffy antigen expression, which could have affected their susceptibility to *P*. *vivax*. Estimated Duffy antigen positivity in Kenya is 5%–10% (*7*). *P*. *vivax* infections in Duffy-negative subjects have been documented in Africa (*2*). Characterization of Duffy antigen expression will be needed to understand the threat of *P*. *vivax* infections in Kenya.

*Anopheles stephensi* mosquitoes have been identified in Kenya (E.O. Ochomo et al., unpub. data, https://doi.org/10.21203/rs.3.rs-2498485/v1), and the potential expansion of this highly competent vector, which survives in urban and manmade habitats, could dramatically change malaria transmission patterns. Continued spread of this invasive vector into sub-Saharan Africa would place ≈126 million persons at risk for malaria (*8*). Identification of *An. stephensi* mosquitoes in Djibouti was linked with a >100-fold rise in malaria cases, including the first autochthonous cases of *P. vivax* reported in 2016 (*9*). 

In conclusion, if emerging *An. stephensi* mosquitoes become established across Kenya in the presence of confirmed *P*. *vivax* cases, malaria elimination in Kenya will be substantially more difficult to achieve. Enhanced surveillance for both *An*. *stephensi* mosquitoes and *P*. *vivax* will be needed to inform control measures, and increased clinical resource allocation will enable detection and effective treatment of patients with *P*. *vivax* malaria.

Appendix Additional information for *Plasmodium* *vivax* prevalence in semiarid region of northern Kenya, 2019.
