# Supplementary material for: Plasmodium vivax Prevalence in Semiarid Region of Northern Kenya, 2019
Source: Emerg Infect Dis. 2023 Nov;29(11):2385–7. doi: 10.3201/eid2911.230299 (PMC10617362; doi:10.3201/eid2911.230299)
Supplement: Appendix — Additional information for Plasmodium vivax prevalence in semiarid region of northern Kenya, 2019. [file 23-0299-Techapp-s1.pdf]

Article DOI: https://doi.org/10.3201/eid2911.230299

EID cannot ensure accessibility for supplementary materials supplied by authors. Readers who have difficulty accessing supplementary content should contact the authors for assistance.

Plasmodium vivax Prevalence in Semiarid Region of Northern Kenya, 2019

Appendix

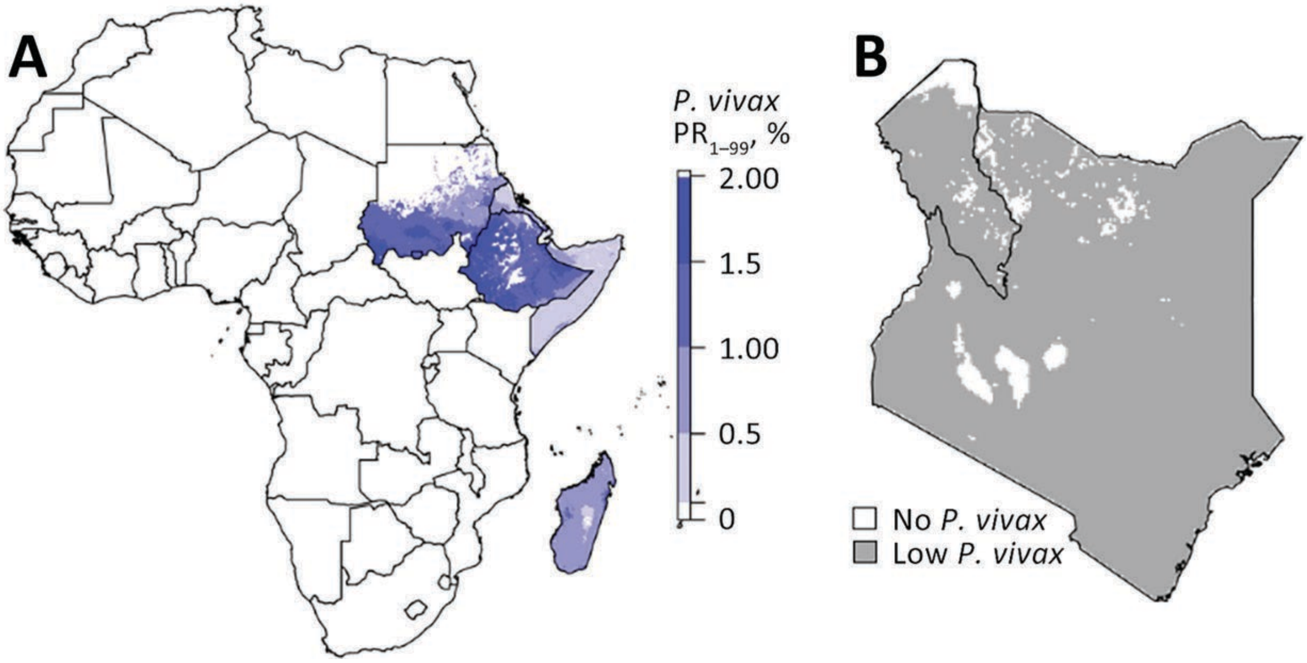

Appendix Figure 1. Estimates of *Plasmodium vivax*–endemic areas across Africa. A) Purple color indicates % prevalence of *P. vivax* infections in Sudan, Ethopia, Eritrea, Somalia, and Madagascar. Ethiopia had established *P. vivax* transmission with a population prevalence rate up to 2%. B) Gray color indicates <0.01% prevalence of *P. vivax* infections in Kenya; transmission is not excluded by climate but is very rare or has not been detected. Turkana county study area in northwestern Kenya is outlined. Data are from the Malaria Atlas Project (https://www.malariaatlas.org). PR, prevalence rate.

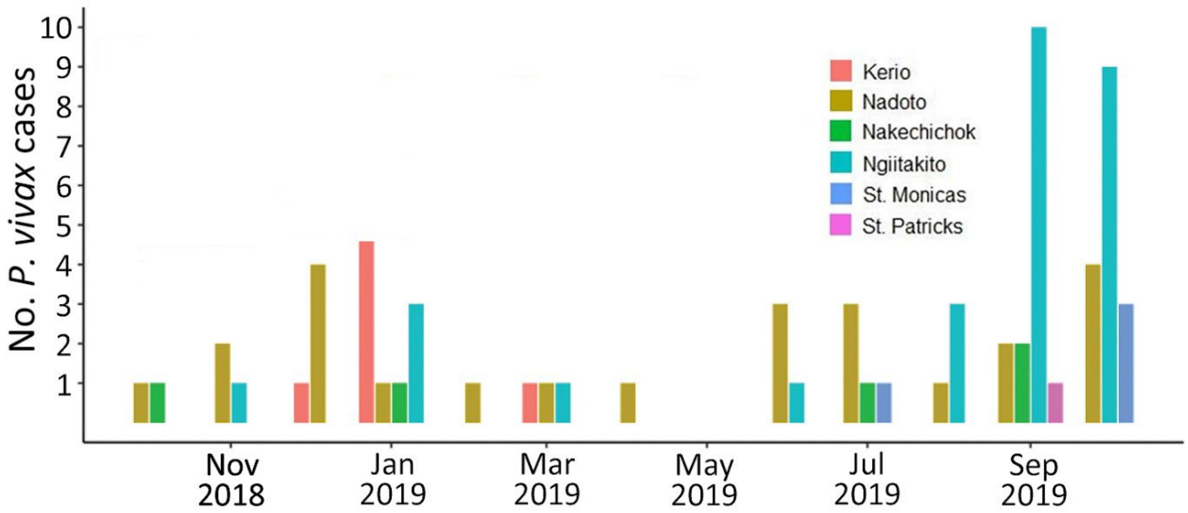

Appendix Figure 2. Number of cases of *Plasmodium vivax* infection according to month and health facility catchment in central Turkana county, Kenya. Household members of patients with *P. falciparum* infections were enrolled in catchment areas surrounding 3 rural and 3 urban health facilities. Colors indicate locations of the health facilities in Turkana county.
